# Factors influencing pathological complete response following neoadjuvant chemoimmunotherapy in locally advanced microsatellite stable colorectal cancer: a retrospective analysis

**DOI:** 10.3389/fmed.2025.1587684

**Published:** 2025-06-06

**Authors:** Mingxiang Liu, Qi Zhou, Bin Lan, Jialiang Gan, Sen Zhang

**Affiliations:** ^1^Department of Colorectal and Anal Surgery, The First Affiliated Hospital, Guangxi Medical University, Nanning, Guangxi, China; ^2^Guangxi Key Laboratory of Enhanced Recovery After Surgery for Gastrointestinal Cancer, Nanning, Guangxi, China

**Keywords:** colorectal cancer, microsatellite stable, proficient mismatch repair, neoadjuvant chemoimmunotherapy, pathological complete response, clinical factors

## Abstract

**Objective:**

The purpose of this study is to identify and analyze the factors associated with achieving pathological complete response (pCR) in patients with locally advanced microsatellite stable (MSS) or proficient mismatch repair (pMMR) colorectal cancer who underwent neoadjuvant chemoimmunotherapy.

**Methods:**

A retrospective cohort study was conducted on 116 such patients at the First Affiliated Hospital of Guangxi Medical University from January 2023 to July 2024. Univariate and multivariate analyses were used to find factors associated with pCR. Perform Cohen’s Kappa coefficient analysis to assess the agreement between clinical staging and pathological staging following neoadjuvant chemoimmunotherapy.

**Results:**

Among 116 patients who received neoadjuvant chemoimmunotherapy followed by radical curative surgery, 32.8% (38/116) achieved a pCR. Univariate analysis showed that pCR was not associated with sex, tumor location, etc., (*P* > 0.05 for all), but was significantly associated with age, body mass index (BMI), neutrophil–lymphocyte ratio (NLR), systemic inflammation index (SII), albumin–to–globulin ratio (AGR), post–treatment carcinoembryonic antigen (CEA) levels, and natural killer (NK) cell count (*P* < 0.05). Multivariate analysis identified age (OR = 0.952, 95% CI: 0.911–0.995, *P* = 0.031) and sex (OR = 0.188, 95% CI: 0.057–0.625, *P* = 0.006) as independent predictors of pCR in locally advanced MSS or pMMR colorectal cancer. Kappa concordance tests indicated poor agreement between post–treatment clinical and pathological staging. The kappa value for clinical T staging versus pathological T staging was 0.006 (*P* = 0.823), and for clinical N staging versus pathological N staging was 0.187 (*P* < 0.001), with a concordance rate of 40.5% (47/116). Stratified by tumor location, in rectal cancer, clinical N staging had moderate agreement with pathological N staging (kappa = 0.273, *P* = 0.004, concordance rate 54.5%, 18/33), while in colon cancer, clinical T staging had negligible agreement (kappa = −0.006, *P* = 0.915), and clinical N staging had low concordance (kappa = 0.154, *P* = 0.001, concordance rate 36.1%, 30/83).

**Conclusion:**

Younger male patients demonstrated a significantly higher likelihood of achieving pCR following neoadjuvant chemoimmunotherapy. This study emphasizes the need to enhance the accuracy of clinical restaging after neoadjuvant chemoimmunotherapy.

## Introduction

Colorectal cancer (CRC) is one of the most prevalent malignancies worldwide, ranking as the third most commonly diagnosed cancer and the second leading cause of cancer-related deaths globally. According to the latest epidemiological data, over 1.9 million new cases and approximately 935,000 deaths were reported in 2020, highlighting the significant burden of this disease on public health (1). The incidence of CRC varies geographically, with higher rates observed in developed regions, but its prevalence is rapidly increasing in developing countries due to lifestyle changes and aging populations (2). Despite advancements in early detection and treatment, a substantial proportion of patients are diagnosed with locally advanced or metastatic disease, which poses significant challenges for effective management (3).

In recent years, immunotherapy has emerged as a transformative approach in cancer treatment, particularly for malignancies with high microsatellite instability (MSI-H) or mismatch repair deficiency (dMMR) (4). Immune checkpoint inhibitors (ICIs), such as anti-PD-1 and anti-CTLA-4 antibodies, have demonstrated remarkable efficacy in MSI-H/dMMR CRC, leading to durable responses and improved survival outcomes (5, 6). However, the majority of CRC cases are characterized by microsatellite stability (MSS) or proficient mismatch repair (pMMR), which are generally considered less responsive to immunotherapy (7, 8). This has spurred extensive research into strategies to overcome the inherent resistance of MSS/pMMR CRC to immune-based therapies (9–11).

Current treatment strategies for MSS CRC have largely focused on conventional chemotherapy, targeted therapies, radiotherapy, and their combinations (12–14). However, the limited efficacy of these approaches in advanced stages has prompted investigations into the potential of combining chemotherapy with immunotherapy. Preclinical and clinical studies suggest that chemotherapy may enhance the immunogenicity of MSS tumors by inducing immunogenic cell death, increasing tumor antigen presentation, and modulating the tumor microenvironment (15–17). These mechanisms provide a rationale for exploring the synergistic effects of chemotherapy and immunotherapy in MSS CRC.

Despite these promising developments, the clinical outcomes of combining chemotherapy with immunotherapy in MSS CRC remain heterogeneous, and the factors influencing treatment efficacy are not fully understood (18). Previous studies have identified tumor-infiltrating lymphocytes (TILs), tumor mutational burden (TMB), and gut microbiome composition as predictive biomarkers for the efficacy of ICIs (19–21). Recent advances in predictive biomarker development have highlighted the importance of multiomics integration for optimizing immunotherapy stratification. Notably, Ye et al. (22) recently developed an integrated Machine Learning and Genetic Algorithm-driven Multiomics analysis framework (iMLGAM), which demonstrates superior performance in forecasting ICIs outcomes compared to conventional single-omics biomarkers. To identify patient subgroups most likely to derive clinical benefit from neoadjuvant chemoimmunotherapy, we conducted a retrospective analysis of data from patients with locally advanced MSS CRC who underwent neoadjuvant chemotherapy combined with immunotherapy at our institution between 2023 and 2024. By analyzing clinical and pathological data, we seek to uncover potential predictors of response to neoadjuvant chemoimmunotherapy in locally advanced MSS or pMMR CRC. Our findings may contribute to the development of personalized treatment approaches and inform future clinical trials in this challenging subset of CRC patients.

## Data and methods

### Study population and data collection

This cohort study analyzed the clinical and pathological data of 155 patients diagnosed with colorectal cancer who underwent immunotherapy at the Department of Colorectal and Anal Surgery, First Affiliated Hospital of Guangxi Medical University, between January 2023 and July 2024. After excluding 29 cases with distant metastasis and 10 cases who did not return to our hospital for surgical treatment, a total of 116 patients were included in the final analysis. Among the included patients, 69 were male and 47 were female, with a median age of 58 years (range: 25–84 years).

The study population consisted of patients with locally advanced MSS or pMMR CRC who received neoadjuvant chemotherapy combined with immunotherapy followed by radical resection. Detailed clinical and pathological data, including demographic characteristics, tumor staging, treatment regimens, and postoperative outcomes, were collected for analysis. This study was approved by the Institutional Review Board of Guangxi Medical University, and informed consent was obtained from all participants.

### Inclusion and exclusion criteria

Inclusion Criteria: (1) Patients aged 18 years or older. (2) Pathologically confirmed adenocarcinoma and confirmed as MSS or pMMR by polymerase chain reaction-capillary electrophoresis (PCR-CE) or immunohistochemistry (IHC). (3) Complete pre-treatment imaging studies with clinical staging of T3-T4 and/or N+ and M0. (4) Receipt of at least one cycle of neoadjuvant chemoimmunotherapy, followed by radical surgical resection. (5) Availability of complete clinical and pathological data for evaluation. Exclusion Criteria: (1) Presence of distant metastasis detected by contrast-enhanced CT scans of the chest, abdomen, and pelvis. (2) Failure to return to our center for surgical treatment or absence of surgical intervention. (3) Incomplete clinical or pathological data.

### Observational parameters

The primary observational indicators in our study included demographic and clinical characteristics such as gender, age, body mass index (BMI), serum total protein concentration, albumin concentration, globulin concentration, albumin-to-globulin ratio (AGR), and systemic inflammatory markers such as the platelet-to-lymphocyte ratio (PLR), lymphocyte-to-monocyte ratio (LMR), neutrophil-to-lymphocyte ratio (NLR), systemic immune-inflammation index (SII), high-sensitivity C-reactive protein (hs-CRP) concentration, and immunoglobulin (IgG, IgA, IgM) concentrations. Additionally, tumor markers (CEA) was assessed before and after neoadjuvant therapy, along with absolute counts of peripheral blood T lymphocytes, CD4+ T lymphocytes, CD8+ T lymphocytes, NK cells, and B lymphocytes. Tumor characteristics, including the longest diameter on imaging before and after neoadjuvant therapy, tumor location (right-sided or left-sided), and tumor differentiation degree, were also evaluated.

### Treatment protocol

All patients were recommended to undergo 4 cycles of neoadjuvant chemoimmunotherapy with the CAPOX regimen combined with anti-PD-1 therapy (oxaliplatin 130 mg/m^2^ every 21 days; capecitabine 1,000 mg/m^2^ twice daily from day 1 to day 14; and tislelizumab 200 mg every 21 days). Following the completion of neoadjuvant therapy, treatment response was evaluated using contrast-enhanced CT and MRI. If R0 resection was deemed achievable, patients proceeded to surgical intervention. If R0 resection was not feasible, the same treatment regimen was continued until R0 resection could be accomplished.

### Neoadjuvant chemoimmunotherapy efficacy assessment

Pathological complete response (pCR) is defined as the absence of cancer cells in both the primary tumor site and regional lymph nodes within the surgically resected specimens following neoadjuvant chemoimmunotherapy. The Ryan/Tumor Regression Grade (TRG) system from the 7th edition of the American Joint Committee on Cancer (AJCC) was utilized to assess the degree of tumor regression after neoadjuvant treatment. In this system, TRG 0 represents a state where there are no cancer cell remnants. TRG 1 indicates that only single cancer cells or small foci of cancer cells are detected as residues. For TRG 2, although there is tumor residue present, its amount is less than that of the fibrotic stroma. And TRG 3 is characterized by extensive tumor residue with little to no cancer cell necrosis.

### Statistical analysis

For quantitative data that conforms to a normal or approximately normal distribution between groups, the *t*-test was used for comparison. For skewed data, it was expressed as the median (interquartile range) [M (P25–P75)], and the Mann–Whitney U test was applied. For count data, Pearson’s chi–square test, or Fisher’s exact test was used for analysis. Binary logistic regression was used to evaluate multiple predictors. The kappa value test was used to assess the consistency between the clinical stage after neoadjuvant chemoimmunotherapy and the postoperative pathological stage. Data analysis was performed using SPSS 26.0 software, and a *P*-value < 0.05 was considered statistically significant.

## Results

### Treatment effect

A total of 116 patients who underwent neoadjuvant chemoimmunotherapy received curative surgery, with all procedures being radical. Postoperative pathological evaluation revealed that 38 patients (32.8%) achieved TRG grade 0 (pathological complete response, pCR), while 78 patients (67.2%) did not attain pCR, including 8 cases of TRG grade 1, 35 cases of TRG grade 2, and 35 cases of TRG grade 3. Additionally, postoperative pathological staging demonstrated statistically significant downstaging in both pathological T stage and N stage (Table 1) compared to pretreatment clinical staging (*P* < 0.001), highlighting the efficacy of neoadjuvant chemoimmunotherapy in reducing tumor and nodal burden. Radiographic measurements of tumor maximum diameter before and after treatment further confirmed significant tumor volume reduction (*P* < 0.001, Table 1). Moreover, comparative analysis of carcinoembryonic antigen (CEA) levels before and after treatment showed a marked decline post-therapy (*P* < 0.001, Table 1). These findings collectively underscore the robust therapeutic effectiveness of neoadjuvant chemoimmunotherapy, as evidenced by enhanced pathological response rates, substantial tumor downstaging, volumetric regression, and reduced serum tumor marker levels.

**TABLE 1:** Comparison of T stage, N stage, tumor diameter, and CEA levels before and after neoadjuvant chemoimmunotherapy.

Variable	Before neoadjuvant chemoimmunotherapy	After neoadjuvant chemoimmunotherapy/after surgery	χ^2^/z	*P*
T stage (*n*)			80.26	< 0.001
T0	0	39		
T1	0	1		
T2	0	9		
T3	92	58		
T4	24	10		
N stage (*n*)			125.56	< 0.001
N0	8	87		
N1	31	22		
N2	77	7		
Tumor maximum diameter (cm)	6.5 (4.9–8.4)	4.6 (3.0–6.0)	−7.708	< 0.001
CEA (ng/ml)	6.71 (2.51–17.65)	3.26 (2.22–5.61)	−5.35	< 0.001

### Pathological complete response and clinicopathological factors

Univariate analysis revealed that pCR following neoadjuvant chemoimmunotherapy was not associated with sex, tumor location, tumor differentiation, pretreatment tumor size, PLR, LMR, hs-CRP, serum total protein, albumin, globulin levels, pretreatment CEA levels, IgG, IgA, IgM levels, peripheral blood T-lymphocyte count, CD4+ T-cell count, CD8+ T-cell count, or B-cell count (*P* > 0.05 for all). However, age, BMI, NLR, SII, AGR, post treatment CEA levels, and peripheral blood natural killer (NK) cell count (*P* < 0.05 for all) exhibited significant associations with pCR achievement (Table 2).

**TABLE 2:** Univariate analysis of pCR and clinicopathological factors.

Variable	pCR group (*n* = 38)	Not pCR group (*n* = 78)	χ^2^/z	*P*
Age (year)	51 (25–69)	60 (32–84)	−3.146	0.002
Sex			3.139	0.076
Male	27	42		
Female	11	36		
Tumor location			0.014	0.91
Right	17	34		
Left	21	44		
Degree of differentiation				
High	7	5	4.57	0.102
Middle	28	61		
Low	3	12		
Longest diameter before therapy (cm)	6.6 (5.5–9.1)	6.5 (4.6–7.9)	−1.348	0.178
PLR	226.1 (161.8–315.6)	188.6 (138.7–267.2)	−1.865	0.062
LMR	2.44 (2.05–3.71)	3.09 (2.61–4.29)	−1.747	0.081
NLR	3.55 (2.59–4.37)	2.61 (1.88–3.28)	−2.512	0.012
SII	1,147.9 (597.3–1,589.7)	838.2 (597.3–1,328.9)	−2.124	0.034
Tpc (g/L)	71.0 (65.6–75.0)	70.3 (65.5–73.7)	−0.541	0.588
Alb (g/L)	38.5 (34.9–41.3)	39.1 (36.4–41.1)	−0.921	0.357
Glb (g/L)	32.6 (28.9–37.1)	31.0 (27.9–33.7)	−1.765	0.078
AGR	1.2 (1.0–1.3)	1.3 (1.2–1.4)	−1.998	0.046
hs-CRP (mg/L)	10.4 (2.9–18.8)	3.6 (1.8–12.9)	−1.759	0.079
Pre-CEA (ng/mL)	7.03 (2.14–20.5)	5.69 (2.58–17.3)	−0.353	0.724
Post-CEA (ng/mL)	2.95 (1.99–4.34)	3.64 (2.35–7.27)	−2.347	0.019
BMI	21.2 (19.1–24.1)	22.4 (20.7–25.17)	−2.032	0.042
T lymphocyte count (cells/μL)	1,132 (885–1,175)	1,186 (959–21,635)	−0.303	0.762
CD4+ T cell (cells/μL)	730 (459–963)	711 (543–963)	−0.015	0.988
CD8+ T cell (cells/μL)	394 (269–588)	398 (298–557)	−0.009	0.993
NK cell (cells/μL)	13.4 (8.3–17.7)	16.3 (10.1–23.1)	−2.009	0.045
B cell (cells/μL)	219 (145–340)	253 (117–355)	−0.015	0.988
IgG (g/L)	13.2 (12–16)	12.7 (11–15)	−1.453	0.146
IgM (g/L)	1.08 (0.84–1.56)	1.06 (0.65–1.30)	−1.927	0.054
IgA (g/L)	2.10 (1.64–2.65)	2.44 (1.83–3.22)	−1.2	0.230

pCR, pathological complete response; PLR, platelet-to-lymphocyte ratio; LMR, lymphocyte-to-monocyte ratio; NLR, neutrophil-to-lymphocyte ratio; SII, systemic immune-inflammation index; Tpc, total protein concentration; Alb, albumin concentration; Glb, globulin concentration; AGR, albumin-to-globulin ratio; hs-CRP, high-sensitivity C-reactive protein; Pre-CEA, pre-treatment carcinoembryonic antigen levels; Post-CEA, post-treatment carcinoembryonic antigen levels; BMI, body mass index.

In multivariate logistic regression analysis, age (odds ratio [OR] = 0.952, 95% confidence interval [CI]: 0.911–0.995, *P* = 0.031) and sex (OR = 0.188, 95% CI: 0.057–0.625, *P* = 0.006) were identified as independent predictors of pCR after neoadjuvant chemoimmunotherapy in locally advanced MSS or pMMR colorectal cancer (Table 3). These findings suggest that younger age and male may serve as favorable prognostic factors for achieving pCR.

**TABLE 3:** Multivariate analysis of pCR after neoadjuvant chemoimmunotherapy for colorectal cancer.

Variable	b value	SE value	Wald value	OR	95% CI	*P*-value
Sex (female)	−1.671	0.612	7.446	0.188	0.057–0.625	0.006
Age	−0.049	0.023	4.677	0.952	0.911–0.995	0.031
PLR	−0.004	0.004	0.791	0.996	0.988–1.005	0.37
LMR	−0.006	0.212	0.001	0.994	0.656–1.505	0.98
NLR	0.503	0.284	3.150	1.654	0.949–2.885	0.076
SII	0.000	0.001	0.019	1.000	0.999–1.001	0.89
Glb	−0.081	0.094	0.738	0.922	0.766–1.110	0.39
AGR	−4.064	2.088	3.788	0.017	0.000–1.029	0.052
hs-CRP	−0.028	0.016	2.959	0.972	0.941–1.004	0.085
Post-CEA	−0.137	0.079	2.978	0.872	0.746–1.019	0.084
BMI	−0.168	0.098	2.939	0.845	0.698–1.024	0.086
NK cell	0.001	0.002	0.716	1.001	0.998–1.005	0.40
IgM	0.862	0.468	3.397	2.369	0.947–5.925	0.065

pCR, pathological complete response; PLR, platelet-to-lymphocyte ratio; LMR, lymphocyte-to-monocyte ratio; NLR, neutrophil-to-lymphocyte ratio; SII, systemic immune-inflammation index; Glb, globulin concentration; AGR, albumin-to-globulin ratio; hs-CRP, high-sensitivity C-reactive protein; Post-CEA, post-treatment carcinoembryonic antigen levels; BMI, body mass index.

Restricted cubic spline (RCS) analysis revealed no significant non-linear association between age and the likelihood of achieving pCR (non-linearity test: *P* = 0.536, Supplementary Figure 1). Instead, a consistent linear trend was observed, with the probability of pCR decreasing progressively as age increased. To further validate this pattern, patients were stratified into three age groups: < 40 years, 40–60 years, and > 60 years. Multivariable logistic regression adjusted for sex demonstrated a stepwise reduction in pCR rates across advancing age categories. Specifically, compared to the < 40-year group, patients aged 40–60 years exhibited a 69.9% lower odds of achieving pCR (adjusted odds ratio [aOR] = 0.301, 95% CI: 0.098–0.918, *P* = 0.035), while those > 60 years showed an 81.6% reduction (aOR = 0.184, 95% CI: 0.058–0.580, *P* = 0.004) (Supplementary Table 1). These findings collectively support a monotonic inverse relationship between age and pCR probability, independent of non-linear effects.

### Comparison of the concordance between clinical staging after neoadjuvant chemoimmunotherapy and postoperative pathological staging

To evaluate the concordance between clinical T and N staging after neoadjuvant chemoimmunotherapy and postoperative pathological staging, we performed kappa concordance tests. The results demonstrated poor agreement between post-treatment clinical staging and pathological staging. Specifically, clinical T staging after neoadjuvant therapy showed almost no concordance with pathological T staging (kappa = 0.01, *P* = 0.823), though the result lacked statistical significance (Table 4). Similarly, the agreement between clinical N staging and pathological N staging was low (kappa = 0.187, *P* < 0.001), with a concordance rate of 40.5% (47/116) (Table 5). Given that clinical staging for rectal cancer primarily relies on pelvic high-resolution MRI and for colon cancer on abdominal contrast-enhanced CT, we further stratified the analysis by tumor location.

**TABLE 4:** Cohen’s κ coefficient for T-stage concordance between clinical and pathological evaluations after neoadjuvant chemoimmunotherapy.

Pathological staging	Clinical T stage (*n*)				Total	Kappa	*P*
	**T0**	**T2**	**T3**	**T4**			
T0	2	1	30	5	38	0.01	0.823
T1	0	0	1	0	1		
T2	0	0	8	1	9		
T3	0	0	47	11	58		
T4	0	1	8	1	10		
Total	2	2	94	18	116		

**TABLE 5:** Cohen’s κ coefficient for N-stage concordance between clinical and pathological evaluations after neoadjuvant chemoimmunotherapy.

Pathological staging	Clinical N stage (*n*)			Total	Kappa	*P*
	**N0**	**N1**	**N2**			
N0	27	24	36	87	0.187	< 0.001
N1	1	13	8	22		
N2	0	0	7	7		
Total	28	37	51	116		

In rectal cancer, clinical T staging post-neoadjuvant therapy exhibited poor concordance with pathological T staging, though this discrepancy was not statistically significant, potentially attributable to random variability (Table 6). Clinical N staging in rectal cancer showed moderate agreement (kappa = 0.273, *P* = 0.004), with a concordance rate of 54.5% (18/33) (Table 7). For colon cancer, a similar pattern of discordance was observed: clinical T staging demonstrated negligible agreement (kappa = −0.006, *P* = 0.915), while clinical N staging exhibited low concordance (kappa = 0.154, *P* = 0.001), with a concordance rate of 36.1% (30/83) (Tables 8, 9).

**TABLE 6:** Cohen’s κ analysis of T-stage concordance between clinical and pathological staging in rectal cancer after neoadjuvant chemoimmunotherapy.

Pathological staging	Clinical T stage (*n*)			Total	Kappa	*P*
	**T2**	**T3**	**T4**			
T0	1	8	2	11	0.044	0.500
T2	0	3	0	3		
T3	0	16	1	17		
T4	0	2	0	2		
Total	1	29	3	33		

**TABLE 7:** Cohen’s κ analysis of N-stage concordance between clinical and pathological staging in rectal cancer after neoadjuvant chemoimmunotherapy.

Pathological staging	Clinical N stage (*n*)			Total	Kappa	*P*
	**N0**	**N1**	**N2**			
N0	10	4	10	24	0.273	0.004
N1	1	7	1	9		
Total	11	11	11	33		

**TABLE 8:** Cohen’s κ analysis of T-stage concordance between clinical and pathological staging in colon cancer after neoadjuvant chemoimmunotherapy.

Pathological staging	Clinical T stage (*n*)				Total	Kappa	*P*
	**T0**	**T2**	**T3**	**T4**			
T0	2	0	22	3	27	−0.006	0.915
T1	0	0	1	0	1		
T2	0	0	5	1	6		
T3	0	0	31	10	41		
T4	0	1	6	1	8		
Total	2	1	65	15	83		

**TABLE 9:** Cohen’s κ analysis of N-stage concordance between clinical and pathological staging in colon cancer after neoadjuvant chemoimmunotherapy.

Pathological staging	Clinical N stage (*n*)			Total	Kappa	*P*
	**N0**	**N1**	**N2**			
N0	17	20	26	63	0.154	0.001
N1	0	6	7	13		
N2	0	0	7	7		
Total	17	26	40	83		

Collectively, these findings indicate suboptimal alignment between post-neoadjuvant clinical staging and pathological staging, predominantly characterized by clinical overstaging. This discrepancy poses significant challenges for clinical decision-making, particularly in determining the necessity of adjuvant therapies or surgical margins. The observed discordance underscores the limitations of conventional imaging modalities in accurately reflecting pathological tumor regression following neoadjuvant chemoimmunotherapy, emphasizing the need for improved staging criteria or multimodal assessment strategies.

## Discussion

In this study, we investigated 116 patients who underwent neoadjuvant chemoimmunotherapy followed by radical surgery for locally advanced MSS or pMMR colorectal cancer. The key findings revealed that 32.8% (38 cases) achieved pCR, while 67.2% (78 cases) did not, with distinct TRG grade distributions. Post-treatment, significant downstaging in both pathological T and N stages, along with marked reductions in tumor maximum diameter and CEA levels, were observed, firmly demonstrating the efficacy of neoadjuvant chemoimmunotherapy. Univariate analysis demonstrated that pCR was not associated with factors like sex and tumor location but was significantly related to age, BMI, etc. Multivariate analysis ultimately identified age (OR = 0.943, *P* = 0.001) and sex (OR = 0.188, 95% CI: 0.057–0.625, *P* = 0.006) as independent predictors of pCR. The analysis demonstrated a significant inverse linear association between age and the likelihood of achieving pCR, with advancing age correlating with a progressively reduced probability of pCR. This trend persisted even after adjusting for potential confounders, suggesting age as an independent predictor of diminished pCR rates. However, kappa concordance tests indicated poor agreement between post-treatment clinical and pathological staging, with negligible concordance in T staging (kappa = 0.006, *P* = 0.823) and low concordance in N staging (kappa = 0.187, *P* < 0.001). Stratification by tumor location showed moderate N staging agreement in rectal cancer (kappa = 0.273, *P* = 0.004) and negligible T staging agreement in colon cancer (kappa = −0.006, *P* = 0.915).

The association between younger age and a higher likelihood of achieving pCR can be attributed to multiple physiological and pathological mechanisms. Physiologically, younger individuals generally possess a more robust immune system. Their T–lymphocytes, which play a pivotal role in cell–mediated immunity, are more active in recognizing and attacking cancer cells (23–25). Compared with young individuals, the functional changes of dendritic cells in the elderly include migration defects and reduced production of selected cytokines. By correcting the age–related defects in dendritic cells, the ability of CD4 T cells to kill tumor cells can be enhanced (26). Natural killer (NK) cells, another crucial component of the innate immune system, also exhibit higher cytotoxic activity in younger patients, being able to directly lyse tumor cells (27).

However, inconsistent with our results, the literature by Zhu et al. (28) demonstrated that older patients with cancers showed enhanced response to ICIs therapy. Through a meta-analysis of 25 studies involving ICIs therapy, they found that the percentages of patients with durable clinical benefit and overall response rate increased with age, and older patients had higher response rates. This discrepancy may be attributed to differences in treatment modalities, patient populations, and tumor types. The ICIs therapy in the literature focused on a wide range of cancer types and mainly explored the relationship between age, gut microbiota, and immunotherapy response. Our neoadjuvant chemoimmunotherapy might have distinct mechanisms and patient–specific factors at play. For example, the drugs used in neoadjuvant chemoimmunotherapy, the timing of treatment, and the characteristics of the tumor microenvironment in our study could all contribute to the different outcomes.

Our analysis revealed a notable sex-based disparity in pCR rates following neoadjuvant chemoimmunotherapy for locally advanced colorectal cancer (LACC), with male patients demonstrating a significantly higher likelihood of achieving pCR compared to their female counterparts (OR = 0.188, 95% CI: 0.057–0.625; *P* = 0.006). This observation aligns with emerging evidence suggesting sex-specific variations in immune checkpoint modulation and tumor microenvironment dynamics, which may differentially influence therapeutic efficacy. Emerging research highlights significant gender disparities in immunotherapy outcomes. A meta-analysis by Conforti et al. (29) demonstrated that male patients derive greater clinical benefits from ICIs compared to females. Further supporting this observation, a pooled analysis of real-world studies revealed that immunotherapy significantly improved overall survival (OS) in male hepatocellular carcinoma (HCC) patients (pooled hazard ratio [HR] 0.79; 95% confidence interval [CI] 0.73–0.86), whereas no comparable survival advantage was observed in female counterparts (HR 0.85; 95% CI 0.70 –1.03) (30). The underlying mechanisms driving these sex-specific responses are multifactorial, with hormonal influences and gut microbiota composition being prominent candidates. Notably, estrogen has been implicated in shaping an immunosuppressive tumor microenvironment (TME) (31). Experimental studies indicate that estrogen receptor (ER) signaling promotes the polarization of tumor-associated macrophages (TAMs) toward an immunosuppressive M2-like phenotype, which subsequently induces CD8+ T cell dysfunction (32). This hormonal axis may partially explain the reduced efficacy of ICIs in female patients, as estrogen-driven immune suppression could counteract therapeutic activation of antitumor immunity. Emerging evidence suggests that sex hormones play a pivotal role in shaping the diversity and composition of the gut microbiota. In healthy females, elevated estrogen levels have been positively correlated with increased abundance of *Bacteroidetes* and inversely associated with *Firmicutes* phyla. Conversely, in males, higher testosterone levels exhibit a positive correlation with specific bacterial genera, such as *Ruminococcus* and *Acinetobacter* (33). These sex-specific microbial signatures may contribute to differential immune modulation, as gut microbiota composition is increasingly recognized as a critical determinant of immunotherapy responsiveness (34–36).

These findings underscore the potential clinical relevance of sex as a predictive biomarker for neoadjuvant therapy optimization in LACC. Paradoxically, preclinical studies have demonstrated that male CD8+ T cells exhibit impaired effector functionality and reduced stem-like properties compared to their female counterparts. Mechanistically, androgen receptor (AR) signaling suppresses the activity and stemness of tumor-infiltrating CD8+ T cells in males by modulating epigenetic and transcriptional differentiation programs. This is further supported by preclinical models showing that castration combined with anti-PD-L1 therapy synergistically restricts tumor growth in male mice, suggesting that androgen ablation may enhance T cell-mediated antitumor immunity (37). However, these findings conflict with clinical meta-analyses. A pooled analysis of 23 randomized trials revealed no statistically significant difference in immunotherapy response rates between males and females, challenging the notion of inherent sex-biased efficacy (38). These contrasting observations underscore the complexity of sex-dependent immune biology and emphasize the need for rigorous stratification by hormonal status, tumor microenvironmental features, and microbiota interactions in future translational studies. Resolving these inconsistencies will require integrative analyses leveraging multi-omics approaches to delineate how androgen signaling intersects with immune checkpoints across sexes and disease contexts.

The observed discrepancy between clinical and pathological staging following neoadjuvant chemoimmunotherapy in LACC underscores critical challenges in current staging paradigms and therapeutic response assessment. This inconsistency may arise from multiple factors. First, conventional imaging modalities (e.g., CT, MRI) primarily evaluate tumor size and anatomical changes, which may inadequately capture the biological effects of immunotherapy, such as immune cell infiltration, tumor microenvironment remodeling, or heterogeneous tumor regression patterns (39). Notably, immunotherapy-induced inflammatory responses or fibrosis could mimic residual disease radiologically, leading to overestimation of clinical stage (40). Conversely, pathological staging reflects microscopic residual tumor burden and treatment-induced regression, including immune-mediated tumor cell death or pCR that are undetectable by imaging. This aligns with prior studies demonstrating that immune checkpoint inhibitors may induce “pseudoprogression” or delayed morphological changes, reducing the predictive accuracy of imaging-based staging in immunotherapy contexts (39). Second, the discordance highlights the limitations of relying solely on traditional Tumor Regression Grade (TRG) systems, which were developed for chemotherapy/radiotherapy responses and may not fully encapsulate immune-specific mechanisms, such as tertiary lymphoid structure formation or spatial immune cell distribution. Emerging evidence suggests that immune-related pathological criteria (e.g., immune-related RECIST) (41) or multimodal biomarkers (e.g., ctDNA dynamics) (42) may enhance post-treatment staging accuracy. Clinically, this staging discordance raises questions about the optimal timing for surgical intervention and adjuvant therapy decisions. Patients with significant pathological downstaging despite stable clinical staging might benefit from de-escalated adjuvant regimens, whereas those with minimal pathological response despite radiological “stability” may require intensified follow-up.

These findings advocate for integrating immune-specific pathological evaluation protocols and functional imaging (e.g., PET-based immune tracers, radiomics) into clinical trials to refine response assessment (39). Prospective validation of composite biomarkers and standardized immune-modified staging criteria is warranted to optimize personalized therapeutic strategies in LACC.

This study offers several notable advantages and innovations. Methodologically, we employed a comprehensive approach that integrated pathological evaluation, imaging analysis, and laboratory biomarker detection. This multimodal approach provided a more accurate and comprehensive assessment of the treatment response. The use of kappa concordance tests to evaluate the agreement between clinical and pathological staging represents a more objective method for analyzing the accuracy of staging methods, which is not commonly utilized in all similar studies. In terms of sample characteristics, our study included a relatively large sample size of 116 patients with locally advanced MSS or pMMR colorectal cancer, larger than some previous studies focusing on the same type of cancer and treatment modality (43). The homogeneous patient population enabled more targeted analysis, minimizing the interference of confounding factors from different genetic and disease–stage backgrounds. Moreover, identifying age and sex as independent predictors of pCR is a novel contribution to the field. These factors not only relate to the tumor itself but also reflect the overall physiological state of the patient, thereby broadening the perspective for predicting pCR in colorectal cancer patients receiving neoadjuvant chemoimmunotherapy.

Despite these achievements, our study has certain limitations. The sample size, although relatively large compared to some previous studies, may still be insufficient to fully explore the intricate relationships between all potential factors and pCR. A larger sample size would be beneficial for further validating the independent predictive value of age and sex and for uncovering other potential factors associated with pCR. Additionally, our study mainly focused on short–term treatment responses, such as the achievement of pCR and the concordance between clinical and pathological staging. Long–term follow–up data, including recurrence rate, overall survival, and disease–free survival, were not included. These long–term outcomes are crucial for a more comprehensive evaluation of the effectiveness of neoadjuvant chemoimmunotherapy. Furthermore, there are other factors that were not considered in this study, such as the patient’s lifestyle factors (smoking, alcohol consumption), gut microbiota, and epigenetic modifications. These factors may potentially influence the response to neoadjuvant chemoimmunotherapy and the achievement of pCR.

The theoretical significance of our study lies in the identification of age and sex as independent predictors of pCR, which enriches the theoretical understanding of the factors influencing the response to neoadjuvant chemoimmunotherapy in colorectal cancer. It provides a new theoretical foundation for further exploring the mechanisms of chemoimmunotherapy response and the relationship between the patient’s overall physiological state and tumor response. In clinical practice, these findings can assist clinicians in better predicting the likelihood of pCR in patients with locally advanced MSS or pMMR colorectal cancer. This prediction can guide the selection of treatment strategies. For instance, for patients with a higher probability of achieving pCR, more conservative surgical approaches may be considered, while for those with a lower probability, more aggressive treatment regimens may be explored. Additionally, the emphasis on improving the accuracy of clinical restaging can lead to more precise surgical planning and adjuvant therapy decisions.

Future studies should focus on validating the current findings in larger and more diverse patient populations. Long–term follow–up studies are essential to evaluate the impact of the identified factors on long–term outcomes. Further research should also delve into the underlying mechanisms of how age and sex affect the response to neoadjuvant chemoimmunotherapy. Integrating other potential factors, such as lifestyle and epigenetic factors, into the research framework may offer a more comprehensive understanding of the treatment response in colorectal cancer patients, ultimately leading to the development of more effective treatment strategies.

## Conclusion

Our study identifies younger age and male gender as independent predictors of pCR following neoadjuvant chemoimmunotherapy in LACC. Notably, patients of younger age demonstrated a significantly higher likelihood of achieving pCR, and this association was further pronounced in male patients. These findings suggest that age- and sex-related biological differences, such as variations in immune microenvironment activity or metabolic drug processing, may critically influence therapeutic efficacy. Further investigation into the mechanistic basis of these associations is warranted to optimize patient stratification and personalize neoadjuvant regimens in LACC.

## Data Availability

Publicly available datasets were analyzed in this study. This data can be found here: the datasets generated during and/or analyzed during the current study are available from the corresponding authors on reasonable request.

## Supplementary material

Supplementary Figure 1 Age and pathologic complete response: a linear association with no significant non-linear trend.
