# Supplementary material for: Factors influencing pathological complete response following neoadjuvant chemoimmunotherapy in locally advanced microsatellite stable colorectal cancer: a retrospective analysis
Source: Front Med (Lausanne). 2025 Jun 6;12:1587684. doi: 10.3389/fmed.2025.1587684 (PMC12179079; doi:10.3389/fmed.2025.1587684)
Supplement: Supplementary file 1 [file Table_1.docx]

Table S1. Association between age categories and pathologic complete response adjusted for sex.

| Variable | b value | SE value | Wald value | OR | 95%CI | P value |
| --- | --- | --- | --- | --- | --- | --- |
| Age(<40 years) |  |  | 8.433 |  |  | 0.015 |
| Age(40-60 years) | -1.202 | 0.570 | 4.450 | 0.301 | 0.098-0.918 | 0.035 |
| Age(>60 years) | -1.694 | 0.586 | 8.346 | 0.184 | 0.058-0.580 | 0.004 |
| Sex(female) | -0.901 | 0.456 | 3.909 | 0.406 | 0.166-0.992 | 0.048 |
