# Supplementary figures and images for: Factors influencing pathological complete response following neoadjuvant chemoimmunotherapy in locally advanced microsatellite stable colorectal cancer: a retrospective analysis
Source: Front Med (Lausanne). 2025 Jun 6;12:1587684. doi: 10.3389/fmed.2025.1587684 (PMC12179079; doi:10.3389/fmed.2025.1587684)

# Age and pCR Probability

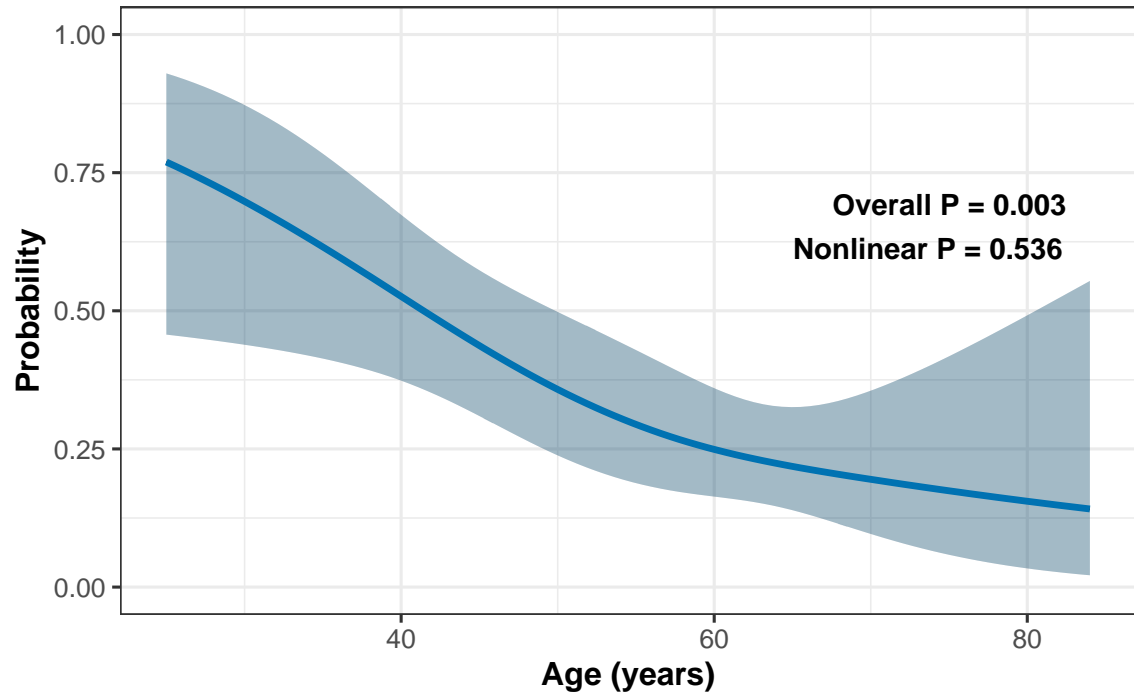

Supplement: Supplementary Figure 1 — Age and pathologic complete response: a linear association with no significant non-linear trend. [file Data_Sheet_1.pdf]
